# Author Correction: Reproductive individuality of clonal fish raised in near-identical environments and its link to early-life behavioral individuality

**DOI:** 10.1038/s41467-026-71983-y

**Published:** 2026-04-13

**Authors:** Ulrike Scherer, Sean M. Ehlman, David Bierbach, Jens Krause, Max Wolf

**Affiliations:** 1 SCIoI Excellence Cluster, Technische Universität Berlin, 10587 Berlin, Germany; 2 Faculty of Life Sciences, Humboldt-Universität zu Berlin, 10117 Berlin, Germany; 3 Department of Fish Biology, Fisheries, and Aquaculture, Leibniz Institute of Freshwater Ecology and Inland Fisheries, 12587 Berlin, Germany

Correction to: *Nature Communications* 10.1038/s41467-023-43069-6, published online 24 November 2023

During the preparation of a follow-up manuscript, we realized that a step in the experimental protocol of this article had not been reported. This previously unreported step does not affect any of the results presented in the original version of this article; we report this step for the sake of experimental reproducibility and full transparency.

In the Methods section of the original version of this article, we reported that individuals were transferred into individual breeding tanks after an initial 28-day behavioral assay phase of the experiment. Unreported, however, was a period of nine days (starting on day 29) between the reported behavioral assay phase and the transfer into individual breeding tanks (where individuals remained till the end of the study, i.e., day 280). During these nine days, individuals were assayed for three behavioral metrics: activity in a new tank, sociability, and response to a novel object. This data was collected (following 3R guidelines to maximize data collected from individual animals) for an auxiliary study that did not bear on the questions addressed in the original manuscript. While the full description and methodological details of these assays are given in the corrected Supplementary Information (Supplementary Note 7: Protocol for the 9-day behavioral assay phase), we here also draw attention to how the findings of our original article remain unaffected by this previously unreported 9-day behavioral assay phase.

In our article, our first and second main results, as described in the Abstract, were that ‘(i) individuals [separated directly after birth into near-identical (i.e., highly standardized) environments] differ consistently in the size of offspring and broods produced over consecutive broods, (ii) these differences are observed even when controlling for trade-offs between brood size, offspring size and reproductive onset, indicating individual differences in life-history productivity.’ The previously unreported nine days of behavioral assays were conducted under highly standardized conditions (see Supplementary Note 7), with all individuals experiencing the same protocol (as in the first 28 days of behavioral observations); thus differences in reproductive outcomes are not systematically affected by the experimental protocol during these nine days (as they are not affected by the experimental protocol of the first 28 days). Our third main result, as listed in the Abstract, reported ‘(iii) early-life behavioral individuality in activity and feeding patterns, with among-individual differences in feeding being predictive of growth, and consequently offspring size.’ The previously unreported nine days do not affect activity and feeding measurements as these were taken beforehand; thus – in combination with the fact that all individuals experienced the same, highly standardized conditions during the additional nine days of behavioral assays – this result also remains unaffected.

The first sentence of the Methods “Reproductive profiles” paragraph has been updated to reflect the 9-day behavioral assay, as has the Supplementary Information. The changes have been made in the HTML and PDF versions of the article.

