# Supplementary material for: Reproductive individuality of clonal fish raised in near-identical environments and its link to early-life behavioral individuality
Source: Nat Commun. 2023 Nov 24;14:7652. doi: 10.1038/s41467-023-43069-6 (PMC10673926; doi:10.1038/s41467-023-43069-6)
Supplement: Supplementary file 1 — Supplementary Information [file 41467_2023_43069_MOESM1_ESM.pdf]

Supplementary information for:

Reproductive individuality of clonal fish raised in near-identical environments and its link to early-life behavioral individuality

Ulrike Scherer[1,2,3]*, Sean M. Ehlman[1,2,3], David Bierbach[1,2,3], Jens Krause[1,2,3] & Max Wolf[1,3]

[1] SCIoI Excellence Cluster, Technische Universität Berlin, 10587 Berlin, Germany
[2] Faculty of Life Sciences, Humbold-Universität zu Berlin, 10117 Berlin, Germany
[3] Department of Fish Biology, Fisheries, and Aquaculture, Leibniz Institute of Freshwater Ecology and Inland Fisheries, 12587 Berlin, Germany

*Corresponding author (u.k.scherer@gmail.com)

CONTENT

CONTENT 2

Supplementary Note 1: Behavioral observations 3

Supplementary Note 2: Food patch preparation 5

Supplementary Note 3: Robustness of results with respect to potential male and/or tank effects 6

Supplementary Note 4: Model output main analyses 12

Supplementary Note 5: Effect of female size on reproductive output 17

Supplementary Note 6: No effects of size at birth on behavior, reproduction, and growth 18

Supplementary Note 7: Protocol for the 9-day behavioral assay phase 21

Supplementary Note 1: Behavioral observations

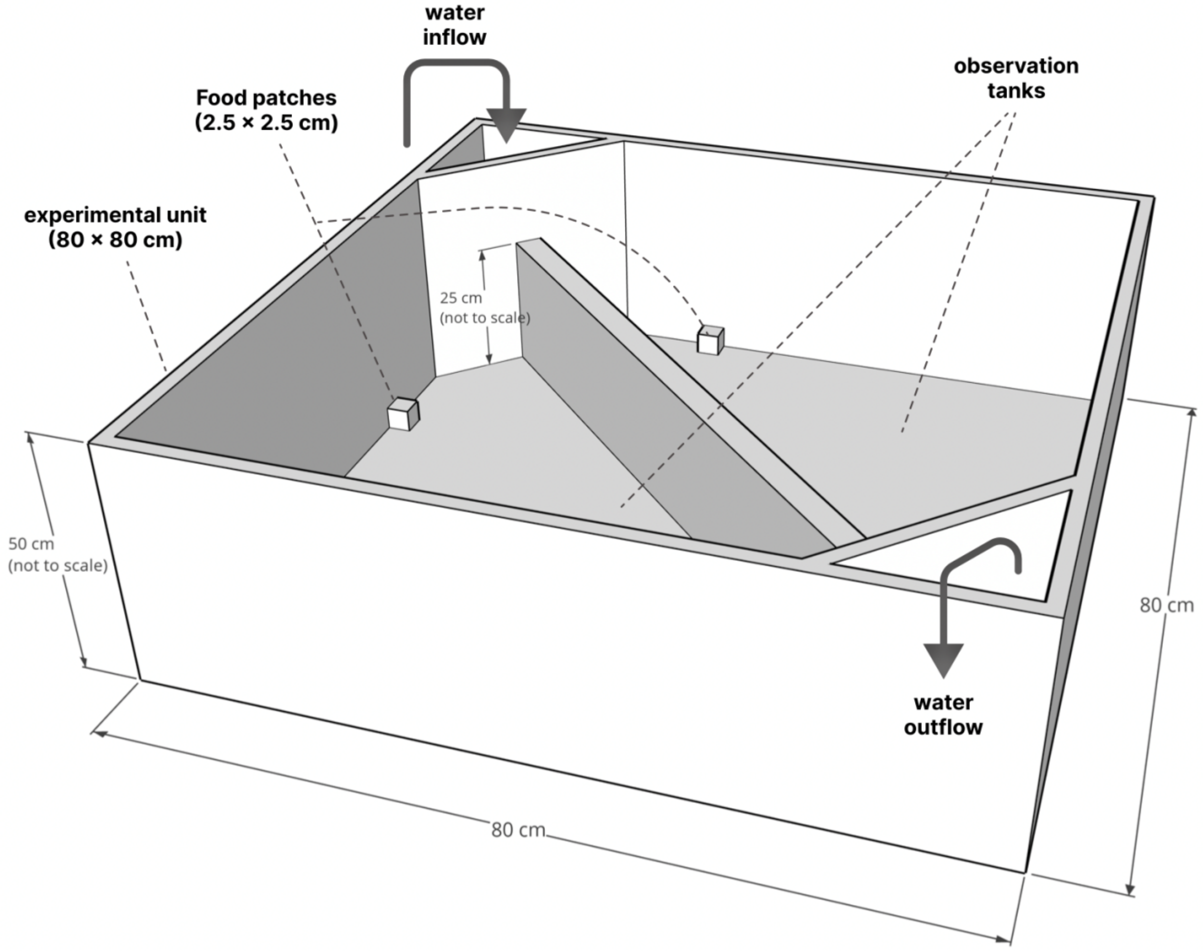

Supplementary Figure 1: Experimental set-up for behavioral observations. One experimental unit with 2 observation tanks. Water level in the tanks: 7 cm. Food patches were present during the feeding only. Observation tanks were illuminated individually from below with 4 LEDs per tank (each LED is 100cm, 12V, color temperature = 5500 K, light output = approx. 1570 lumen; tanks were manufactured from white polyethylene, which allowed light from underneath to get through). There was no visual contact between observation tanks, but tanks were connected via a flow-through water system (24 observation tanks split into 4 flow-through systems). We found no significant effect of the tank system (1-4), distance to the external filter unit (0-2m) or centrality of the tank on individual daily activity or feeding (**Supplementary Table 1**).

Supplementary Table 1 Summaries of linear-mixed effects models testing for potential environmental effects on early-life behavior (activity and feeding). We found no effects of tank system (1-4), distance to the filter (0-2 meters) or centrality (center vs. periphery, referring to a tank's position within a filter system) on individual behavior. Shown are full models (containing all predictor variables; left) and final models (significant predictors only; right).

Response	*Predictor*	Full model			Final model		
		Estimate	*CI*	*P-value*	*Estimate*	*CI*	*P-value*
Activity [cm/sec]	Intercept	0.103	-0.284 – 0.489	-	-0.027	-0.185 – 0.132	-
	Tank system [2]	-0.219	-0.654 – 0.216	0.699	-	-	-
	Tank system [3]	-0.228	-0.653 – 0.196		-	-	-
	Tank system [4]	-0.119	-0.518 – 0.279		-	-	-
	Distance to filter [m]	-0.125	-0.312 – 0.062	0.195	-	-	-
	Centrality [periphery]	0.189	-0.122 – 0.500	0.238	-	-	-
	**Random effects**						
	σ^2	0.37			0.37		
	τ_{00}	0.18 Female ID			0.21 Female ID		
	ICC	0.33			0.36		
	N	34 Female ID			34 Female ID		
	Observations	941			941		
	Marginal R^2 / Conditional R^2	0.051 / 0.363			0.000 / 0.363		
Feeding [min]	Intercept	49.614	38.313 – 60.915	-	48.068	43.127 – 53.008	-
	Tank system [2]	4.877	-7.840 – 17.594	0.091	-	-	-
	Tank system [3]	11.747	-0.661 – 24.156		-	-	-
	Tank system [4]	-3.828	-15.486 – 7.830		-	-	-
	Distance to filter [m]	1.127	-4.340 – 6.595	0.686	-	-	-
	Centrality [periphery]	-8.100	-17.191 – 0.991	0.081	-	-	-
	**Random effects**						
	σ^2	845.77			845.81		
	τ_{00}	134.08 Female ID			184.56 Female ID		
	ICC	0.14			0.18		
	N	34 Female ID			34 Female ID		
	Observations	931			931		
	Marginal R^2 / Conditional R^2	0.049 / 0.179			0.000 / 0.179		

Supplementary Note 2: Food patch preparation

Ingredients

- 0.5g Agar (Bio Agar-Agar, Ruf)
- 100 ml tap water
- 14 g powder food (Sera vipan baby)

Materials

- One fine scale
- One 150 ml beaker
- One small pot
- One stove plate
- One pipette
- Food patch cups (max. volume = 2ml)

Instructions

1. Prepare clean and dry food patch forms.
2. Weigh the amount of agar needed in a 150 ml beaker.
3. Add 100 ml of water.
4. Fill the water-agar mixture into a small pot and cook for 2 min at medium heat.
5. Remove pot from stove and add powder food, mix thoroughly.
6. Let cool down for approx. 5 min at room temperature until agar starts binding.
7. Quickly fill the agar-food mixture into the cups using the pipette.
8. Let the food patches harden in the fridge for approx. 10 min without a lid.
9. Store food patches in a container with a lid (e.g., petri dishes) in the fridge until further proceeding (can be stored for at least 4 days when stored at 4°C).

Note:

The protocol can be modified regarding the amount of food or agar added. Adding more agar will give the mixture a firmer texture. Using less agar is not advised. The amount of agar used here is the least amount possible (resulting in a very soft texture) in order to allow juvenile fish to feed on the food patches. The amount of food can be both decreased and increased allowing for variations in food quality; food variations may require adjustments in the amount of agar used.

Supplementary Note 3: Robustness of results with respect to potential male and/or tank effects

We tested whether our results and conclusions are robust with respect to potential variation between males and/or breeding tanks. In order to do so, we used our data to back-calculate the presumed males that triggered embryonic development/tanks where embryonic development was triggered (in the following: male/tank ID) for all broods (see "Assigning male/tank IDs" below). We then repeated all our analyses involving female reproductive output, controlling for male/tank ID. Importantly, we find that there is no effect of male/tank ID on female reproductive individuality (**Supplementary Table 2**) or its link to early-life behavior (**Supplementary Tables 3-6**), i.e., we find that all our results are robust and qualitatively the same when controlling statistically for potential variation between males and/or breeding tanks.

More specifically, we recalculated normal and adjusted repeatabilities using the same models that we used in the main text (for model structures see main text) except that we now additionally included male/tank ID as a random effect. Including male/tank ID has no effect on female repeatabilities (**Supplementary Table 2**). We note that male/tank ID is associated with repeatable differences in offspring size (but not brood size) (see **Supplementary Table 2**). This finding should be treated with caution with respect to interpreting it as a potential male effect, as our experiment was neither designed to detect potential male effects nor to distinguish between potential male and potential breeding tanks effects but rather to detect female effects while controlling for both male and tank effects.

Similarly, for all other analyses involving brood size or offspring size, we find qualitatively the same results as presented in the main text when including male/tank ID as a random effect: there is a weak brood vs. offspring size trade-off (**Supplementary Table 3**); there is no direct effect of early-life behavior on reproductive output (**Supplementary Table 4**) but larger fish (which feed more) produce larger offspring (**Supplementary Table 5**) (to compare with main text analyses see **Supplementary Table 8**, **Table 9** and **Table 11**). Similarly, we find qualitatively the same results as presented in the main text when testing for a potential effect of female size at birth on reproductive output (**Supplementary Table 6**) (to compare with main text analyses see model summary in **Supplementary Table 13**).

The fact that all our results and conclusions are robust with respect to the above statistical controls of potential effects of males and/or breeding tanks supports the effectiveness of our experimental design, which was aimed at experimentally controlling for both effects of males and breeding tanks simultaneously (see "Reproductive profiles" in the main text), was successful.

Assigning male/tank IDs

Poecilid females typically undergo a reproductive cycle with approx. 2-3 fertile days, which are induced at maturation and immediately after parturition, followed by approx. 30 days during which females are unreceptive to sperm while fertilized eggs are carried internally until parturition [1,2]. For all broods, we thus assigned the male/tank ID to that male/tank a female was with 30 ± 2 days before parturition. If a female was with two different males/in two different tanks within that time frame, we assigned male/tank ID to the male/tank she was with on day 30. If a female was with two different males/in two different tanks on day 30, we assigned male/tank ID to the latter (N = 27 broods).

Because we have low certainty in male/tank ID (*N* = 100 broods with female access to two males/tanks within 30 ± 2 days prior to parturition), we additionally assigned male/tank ID using two alternative methods: first male/tank ID (assigned to the first male/tank a female was 30 ± 2 days prior to parturition) and last male/tank ID (assigned to the second male/tank a female was 30 ± 2 days prior to parturition), accounting, for example, for potential first or last male precedence or deviations from the average gestation period. All analyses presented (**Supplementary Tables 2-6**) were carried out with the "exact" male/tank assignment method but all results are robust with respect to the assignment method, i.e., we repeated all analyses with either the first or last male/tank ID and received qualitatively the same results (not presented here, except **Supplementary Table 2** repeatabilities).

Again, during the reproductive phase of the experiment, females were swapped weekly between breeding tanks, where each breeding tank held a 'resident' male that did not switch tanks (thus tank ID and male ID usually had a one-to-one correspondence). During the experiment, however, some males needed to be replaced, leading to a few incidences where tank ID and male ID were not identical (N = 7 broods). Analyses presented here (**Supplementary Tables 2-6**) were carried out with male ID; however, we repeated all of the above analyses (**Supplementary Tables 2-6**) with tank ID (N = 39 tank where embryonic development was triggered) instead of male ID (N = 45 males that triggered embryonic development) and obtained qualitatively the same results (not presented).

References

1. Farr, J. A. & Travis, J. Fertility Advertisement by Female Sailfin Mollies, *Poecilia latipinna* (Pisces: Poeciliidae). *Copeia* 1986, 467–472 (1986).
2. Snelson, F. F., Wetherington, J. D. & Large, H. L. The Relationship between Interbrood Interval and Yolk Loading in a Generalized Poeciliid Fish, *Poecilia latipinna*. *Copeia* 1986, 295–304 (1986).

Supplementary Table 2 Normal and adjusted repeatabilities (accounting for female size and age, onset, and mother ID) for test fish and male/tank ID (N (brood size) = 152 broods, N (offspring size = 144 broods).

Response	*Method to assign male/tank ID*	*Normal repeatability [CI]*		*Adjusted repeatability [CI]*	
		Female ID	*Male/tank ID*	*Female ID*	*Male/tank ID*
Offspring size [mm]	No male/tank ID	0.396 [0.308, 0.484]	-	0.134 [0.085, 0.192]	-
	Exact ID	0.379 [0.279, 0.473]	0.068 [0.042, 0.110]	0.115 [0.073, 0.170]	0.131 [0.086, 0.187]
	First ID	0.405 [0.307, 0.499]	0.039 [0.025, 0.062]	0.117 [0.074, 0.174]	0.109 [0.073, 0.158]
	Last ID	0.338 [0.243, 0.431]	0.126 [0.085, 0.178]	0.126 [0.082, 0.181]	0.143 [0.098, 0.198]
Brood size	No male/tank ID	0.177 [0.117, 0.238]	-	0.077 [0.050, 0.114]	-
	Exact ID	0.212 [0.141, 0.289]	0.000 [0.000, 0.000]	0.085 [0.052, 0.124]	0.000 [0.000, 0.000]
	First ID	0.198 [0.126, 0.275]	0.015 [0.009, 0.023]	0.087 [0.053, 0.126]	0.000 [0.000, 0.000]
	Last ID	0.210 [0.135, 0.297]	0.000 [0.000, 0.000]	0.084 [0.053, 0.127]	0.000 [0.000, 0.000]

Supplementary Table 3 Full (left) and final (right) linear mixed-effect models testing for an offspring size vs. brood size trade-off, controlling for both the test fish ID and male/tank ID.

		**Full model**			**Final model**		
Response	*Predictor*	*Estimate*	*CI*	*P-value*	*Estimate*	*CI*	*P-value*
Offspring size [mm]	Intercept	5.151	4.333 – 5.968	-	4.540	3.906 – 5.173	-
	Brood size	-0.012	-0.018 – -0.006	<0.001	-0.013	-0.018 – -0.007	<0.001
	Onset [days]	-0.003	-0.007 – 0.001	0.209	-	-	-
	Size at parturition [cm]	0.921	0.739 – 1.104	<0.001	0.970	0.813 – 1.128	<0.001
	Mother ID [m2]	0.026	-0.202 – 0.254	0.078	-	-	-
	Mother ID [m3]	-0.193	-0.364 – -0.022		-	-	-
	**Random effects**						
	σ^2	0.08			0.08		
	τ_{00}	0.01 Male/tank ID			0.01 Male/tank ID		
		0.01 Test fish ID			0.01 Test fish ID		
	ICC	0.22			0.24		
	N	34 Test fish ID			34 Test fish ID		
		45 Male/tank ID			45 Male/tank ID		
	Observations	144			144		
	Marginal R^2 / Conditional R^2	0.583 / 0.675			0.539 / 0.651		

Supplementary Table 4 Full (left) and final (right) linear mixed-effect models testing if early-life behavior (activity, feeding) predicts reproduction (brood size, offspring size, onset), controlling for both test fish ID and male/tank ID.

Response	*Predictor*	**Full model**			**Final model**		
		Estimate	*CI*	*P-value*	*Estimate*	*CI*	*P-value*
Brood size	Intercept	-15.03	-35.58 – 5.53	-	-19.90	-39.82 – 0.03	-
	Activity [cm/sec]	-2.32	-4.88 – 0.25	0.082	-	-	-
	Feeding [min]	-0.07	-0.21 – 0.07	0.312	-	-	-
	Size at parturition [cm]	8.00	3.31 – 12.70	0.001	7.82	3.16 – 12.48	0.001
	Mother ID [m2]	6.11	0.05 – 12.17	<0.001	4.32	-1.61 – 10.26	<0.001
	Mother ID [m3]	10.21	5.71 – 14.71		8.44	4.35 – 12.53	
	**Random effects**						
	σ^2	70.72			70.53		
	τ_{00}	0.00 Male/tank ID			0.00 Male/tank ID		
		7.54 Test fish ID			10.07 Test fish ID		
	N	34 Test fish ID			34 Test fish ID		
		45 Male/tank ID			45 Male/tank ID		
	Observations	152			152		
	Marginal R^2 / Conditional R^2	0.174 / NA			0.155 / NA		
Offspring size [mm]	Intercept	5.176	4.379 – 5.974	-	5.162	4.397 – 5.927	-
	Activity [cm/sec]	-0.039	-0.141 – 0.064	0.459	-	-	-
	Feeding [min]	0.002	-0.004 – 0.007	0.537	-	-	-
	Size at parturition [cm]	0.787	0.607 – 0.967	<0.001	0.799	0.621 – 0.978	<0.001
	Mother ID [m2]	-0.037	-0.284 – 0.209	0.024	-0.086	-0.321 – 0.148	0.014
	Mother ID [m3]	-0.264	-0.453 – -0.076		-0.265	-0.437 – -0.094	
	**Random effects**						
	σ^2	0.09			0.09		
	τ_{00}	0.01 Male/tank ID			0.01 Male/tank ID		
		0.01 Test fish ID			0.02 Test fish ID		
	ICC	0.20			0.22		
	N	34 Test fish ID			34 Test fish ID		
		45 Male/tank ID			45 Male/tank ID		
	Observations	144			144		
	Marginal R^2 / Conditional R^2	0.550 / 0.638			0.539 / 0.642		

Supplementary Table 5 Full (left) and final (right) linear mixed-effect models testing if von Bertalanffy growth parameters (the growth rate K and maximum predicted size L_{inf}) predict reproduction (brood size, offspring size, onset of reproduction), controlling for both test fish ID and male/tank ID.

Response	*Predictor*	Full model			Final model		
		Estimate	*CI*	*P-value*	*Estimate*	*CI*	*P-value*
Brood size	Intercept	43.170	-71.776 – 158.117	-	7.187	1.216 – 13.158	-
	L_{inf} [cm]	-4.576	-23.303 – 14.152	0.630	-	-	-
	K	-173.701	-530.521 – 183.119	0.337	-	-	-
	Age at parturition [weeks]	0.205	0.015 – 0.394	0.036	0.215	0.026 – 0.404	0.027
	Mother ID [m2]	5.630	-0.430 – 11.690	0.113	5.085	-0.828 – 10.999	0.011
	Mother ID [m3]	3.808	-4.004 – 11.620		5.875	2.167 – 9.583	
	**Random effects**						
	**σ^2**	73.73			73.90		
	**τ_{00}**	0.00 Male/tank ID			0.00 Male/tank ID		
		8.36 Test fish ID			9.32 Test fish ID		
	N	34 Test fish ID			34 Test fish ID		
		45 Male/tank ID			45 Male/tank ID		
	Observations	152			152		
	Marginal R^2 / Conditional R^2	0.132 / NA			0.117 / NA		
Offspring size [mm]	Intercept	5.730	1.557 – 9.903	-	4.895	3.475 – 6.315	-
	L_{inf} [cm]	0.472	-0.206 – 1.149	0.175	0.600	0.297 – 0.904	<0.001
	K	-2.777	-15.764 – 10.210	0.674	-	-	-
	Age at parturition [weeks]	0.029	0.022 – 0.037	<0.001	0.029	0.022 – 0.037	<0.001
	Mother ID [m2]	-0.067	-0.288 – 0.154	0.033	-0.079	-0.292 – 0.134	0.002
	Mother ID [m3]	-0.383	-0.668 – -0.098		-0.333	-0.495 – -0.170	
	**Random effects**						
	**σ^2**	0.10			0.10		
	**τ_{00}**	0.00 Male/tank ID			0.00 Male/tank ID		
		0.01 Test fish ID			0.01 Test fish ID		
	ICC	0.11			0.12		
	N	34 Test fish ID			34 Test fish ID		
		45 Male/tank ID			45 Male/tank ID		
	Observations	144			144		
	Marginal R^2 / Conditional R^2	0.580 / 0.628			0.578 / 0.628		

Supplementary Table 6 Full (left) and final (right) linear mixed-effect models testing if size on the first day of life predicts reproductive output, controlling for both test fish ID and presumed male/tank ID.

Response	Predictor	Full model			Final model		
		Estimate	*CI*	*P-value*	*Estimate*	*CI*	*P-value*
Brood size	Intercept	-1.708	-18.717 – 15.301	-	7.187	1.216 – 13.158	-
	Size at birth [cm]	10.904	-8.549 – 30.357	0.274	-	-	-
	Age at parturition [weeks]	0.208	0.019 – 0.398	0.032	0.215	0.026 – 0.404	0.027
	Mother ID [m2]	4.675	-1.168 – 10.518	0.087	5.085	-0.828 – 10.999	0.011
	Mother ID [m3]	4.561	0.254 – 8.869		5.875	2.167 – 9.583	
	**Random effects**						
	σ^2	74.06			73.90		
	τ_{00}	0.00 Male/tank ID			0.00 Male/tank ID		
		8.19 Test fish ID			9.32 Test fish ID		
	N	34 Test fish ID			34 Test fish ID		
		45 Male/tank ID			45 Male/tank ID		
	Observations	152			152		
	Marginal R^2 / Conditional R^2	0.127 / NA			0.117 / NA		
Offspring size [mm]	Intercept	8.076	7.360 – 8.791	-	7.682	7.446 – 7.918	-
	Size at birth [cm]	-0.484	-1.313 – 0.345	0.255	-	-	-
	Age at parturition [weeks]	0.031	0.024 – 0.038	<0.001	0.031	0.024 – 0.038	<0.001
	Mother ID [m2]	0.007	-0.242 – 0.255	<0.001	-0.016	-0.267 – 0.235	<0.001
	Mother ID [m3]	-0.450	-0.644 – -0.257		-0.509	-0.677 – -0.340	
	**Random effects**						
	σ^2	0.09			0.09		
	τ_{00}	0.01 Male/tank ID			0.01 Male/tank ID		
		0.02 Test fish ID			0.02 Test fish ID		
	ICC	0.26			0.27		
	N	34 Test fish ID			34 Test fish ID		
		45 Male/tank ID			45 Male/tank ID		
	Observations	144			144		
	Marginal R^2 / Conditional R^2	0.527 / 0.651			0.520 / 0.648		

Supplementary Note 4: Model output main analyses

Supplementary Table 7 Full (left) and final (right) linear mixed-effect models testing if early-life feeding behavior and activity are correlated.

Response	Predictor	Full model			Final model		
		Estimate	CI	P-value	Estimate	CI	P-value
Feeding [min]	Intercept	55.554	48.433 – 62.675	-	60.734	55.675 – 65.792	-
	Activity [cm/sec]	-9.222	-10.644 – -7.800	<0.001	-9.134	-10.550 – -7.719	<0.001
	Mother ID [m2]	3.103	-11.814 – 18.020	0.088	-	-	-
	Mother ID [m3]	10.495	0.907 – 20.084		-	-	-
	**Random effects**						
	σ^2	721.68			721.69		
	τ_{00}	150.82 Test fish ID			165.52 Test fish ID		
	ICC	0.17			0.19		
	N	34 Test fish ID			34 Test fish ID		
	Observations	931			931		
	Marginal R^2 / Conditional R^2	0.174 / 0.317			0.164 / 0.320		

Supplementary Table 8 Full (left) and final (right) linear mixed-effect models testing for an offspring size vs. brood size trade-off.

Response	*Predictor*	Full model			Final model		
		Estimate	*CI*	*P-value*	*Estimate*	*CI*	*P-value*
Offspring size [mm]	Intercept	5.083	4.238 – 5.927	-	4.466	3.827 – 5.104	-
	Brood size	-0.011	-0.018 – -0.005	<0.001	-0.013	-0.018 – -0.007	<0.001
	Onset [days]	-0.002	-0.006 – 0.002	0.335	-	-	-
	Size at parturition [cm]	0.919	0.730 – 1.108	<0.001	0.988	0.830 – 1.146	<0.001
	Mother ID [m2]	-0.038	-0.273 – 0.196	0.061	-	-	-
	Mother ID [m3]	-0.197	-0.359 – -0.035		-	-	-
	**Random effects**						
	σ^2	0.10			0.10		
	τ_{00}	0.01 Test fish ID			0.01 Test fish ID		
	ICC	0.10			0.13		
	N	34 Test fish ID			34 Test fish ID		
	Observations	144			144		
	Marginal R^2 / Conditional R^2	0.580 / 0.623			0.545 / 0.602		

Supplementary Table 9 Full (left) and final (right) linear mixed-effect models testing if early-life behavior (activity, feeding) predicts reproduction (brood size, offspring size, onset).

Response	*Predictor*	Full model			Final model		
		Estimate	*CI*	*P-value*	*Estimate*	*CI*	*P-value*
Brood size	Intercept	-15.029	-35.583 – 5.524	-	-19898	-39.823 – 0.027	-
	Activity [cm/sec]	-2.317	-4.881 – 0.248	0.082	-	-	-
	Feeding [min]	-0.071	-0.207 – 0.066	0.312	-	-	-
	Size at parturition [cm]	8.002	3.307 – 12.697	0.001	7820	3.157 – 12.484	0.001
	Mother ID [m2]	6.108	0.048 – 12.169	<0.001	4324	-1.608 – 10.256	<0.001
	Mother ID [m3]	10.208	5.706 – 14.710		8444	4.353 – 12.535	
	**Random effects**						
	σ^2	70.72			70.53		
	τ_{00}	7.54 Test fish ID			10.07 Test fish ID		
	ICC	0.10			0.12		
	N	34 Test fish ID			34 Test fish ID		
	Observations	152			152		
	Marginal R^2 / Conditional R^2	0.160 / 0.241			0.138 / 0.246		
Offspring size [mm]	Intercept	5.168	4.359 – 5.978	-	5.147	4.366 – 5.928	-
	Activity [cm/sec]	-0.047	-0.149 – 0.056	0.369	-	-	-
	Feeding [min]	0.002	-0.004 – 0.007	0.513	-	-	-
	Size at parturition [cm]	0.791	0.607 – 0.975	<0.001	0.804	0.622 – 0.987	<0.001
	Mother ID [m2]	-0.058	-0.305 – 0.190	0.018	-0.119	-0.356 – 0.118	<0.001
	Mother ID [m3]	-0.265	-0.446 – -0.084		-0.270	-0.434 – -0.107	
	**Random effects**						
	σ^2	0.10			0.10		
	τ_{00}	0.01 Test fish ID			0.02 Test fish ID		
	ICC	0.12			0.14		
	N	34 Test fish ID			34 Test fish ID		
	Observations	144			144		
	Marginal R^2 / Conditional R^2	0.552 / 0.607			0.539 / 0.605		
Onset of reproduction [days]	Intercept	126.473	94.420 – 158.526	-	133.786	123.545 – 144.027	-
	Activity [cm/sec]	4.000	-6.139 – 14.138	0.426	-	-	-
	Feeding [min]	0.072	-0.458 – 0.602	0.784	-	-	-
	Mother ID [m2]	15.777	-9.037 – 40.590	0.004	19.964	-1.760 – 41.689	0.002
	Mother ID [m3]	-19.908	-35.944 – -3.872		-17.411	-31.434 – -3.387	
	Observations	34			34		
	R^2 / R^2 adjusted	0.341 / 0.250			0.326 / 0.282		

Supplementary Table 10 Full (left) and final (right) linear mixed-effect models testing for an effect of early-life behavior on the maximum predicted size (L_{inf}) and growth rate (K) of individuals.

Response	*Predictor*	Full model			Final model		
		Estimate	*CI*	*P-value*	*Estimate*	*CI*	*P-value*
L_{inf} [cm]	Intercept	4.465	4.159 – 4.771	-	4.375	4.141 – 4.608	-
	Activity [cm/sec]	-0.045	-0.141 – 0.052	0.354	-	-	-
	Feeding [min]	0.006	0.001 – 0.011	0.015	0.007	0.003 – 0.012	0.002
	Mother ID [m2]	0.144	-0.093 – 0.380	<0.001	0.098	-0.116 – 0.313	<0.001
	Mother ID [m3]	-0.349	-0.502 – -0.196		-0.378	-0.517 – -0.239	
	Observations	34			34		
	R^2 / R^2 adjusted	0.592 / 0.535			0.579 / 0.537		
K	Intercept	0.296	0.247 – 0.344	-	0.305	0.263 – 0.346	-
	Activity [cm/sec]	0.001	-0.001 – 0.004	0.326	-	-	-
	Feeding [min]	0.000	-0.000 – 0.000	0.963	-	-	-
	L_{inf} [cm]	-0.046	-0.056 – -0.035	<0.001	-0.047	-0.056 – -0.039	<0.001
	Mother ID [m2]	0.004	-0.003 – 0.010	<0.001	0.005	-0.001 – 0.011	<0.001
	Mother ID [m3]	-0.019	-0.024 – -0.013		-0.018	-0.023 – -0.014	
	Observations	34			34		
	R^2/ R^2 adjusted	0.814 / 0.781			0.807 / 0.788		

Supplementary Table 11 Full (left) and final (right) linear mixed-effect models testing if von Bertalanffy growth parameters (the growth rate K and maximum predicted size L_{inf}) predict reproduction (brood size, offspring size, onset of reproduction).

Response	*Predictor*	Full model			Final model		
		Estimate	*CI*	*P-value*	*Estimate*	*CI*	*P-value*
Brood size	Intercept	43.171	-71.768 – 158.110	-	7.187	1.217 – 13.157	-
	L_{inf} [cm]	-4.576	-23.302 – 14.151	0.630	-	-	-
	K	-173.702	-530.501 – 183.096	0.338	-	-	-
	Age at parturition [weeks]	0.205	0.015 – 0.394	0.035	0.215	0.026 – 0.404	0.026
	Mother ID [m2]	5.630	-0.429 – 11.690	0.111	5.085	-0.828 – 10.999	0.011
	Mother ID [m3]	3.808	-4.004 – 11.620		5.875	2.167 – 9.583	
	**Random effects**						
	σ^2	73.73			73.90		
	τ_{00}	8.36 Test fish ID			9.32 Test fish ID		
	ICC	0.10			0.11		
	N	34 Test fish ID			34 Test fish ID		
	Observations	152			152		
	Marginal R^2 / Conditional R^2	0.120 / 0.210			0.105 / 0.206		
Offspring size [mm]	Intercept	5.739	1.240 – 10.239	-	4.808	3.310 – 6.307	-
	L_{inf} [cm]	0.475	-0.257 – 1.206	0.170	0.619	0.299 – 0.939	<0.001
	K	-3.076	-17.062 – 10.909	0.638	-	-	-
	Age at parturition [weeks]	0.029	0.022 – 0.037	<0.001	0.030	0.022 – 0.037	<0.001
	Mother ID [m2]	-0.081	-0.318 – 0.155	0.026	-0.096	-0.320 – 0.128	<0.001
	Mother ID [m3]	-0.385	-0.689 – -0.080		-0.329	-0.497 – -0.162	
	**Random effects**						
	σ^2	0.10			0.10		
	τ_{00}	0.01 Test fish ID			0.01 Test fish ID		
	ICC	0.12			0.11		
	N	34 Test fish ID			34 Test fish ID		
	Observations	144			144		
	Marginal R^2 / Conditional R^2	0.568 / 0.618			0.570 / 0.616		
Onset of repro-duction [days]	Intercept	-141.259	-595.812 – 313.294	-	-204.285	-383.856 – -24.714	-
	L_{inf} [cm]	45.090	-29.090 – 119.270	0.224	55.537	25.189 – 85.886	0.001
	K	768.659	-631.930 – 2169.249	0.271	980.383	235.137 – 1725.629	0.012
	Mother ID [m2]	15.631	-7.523 – 38.785	0.393	-	-	-
	Mother ID [m3]	-0.368	-31.794 – 31.058		-	-	-
	Observations	34			34		
	R^2 / R^2 adjusted	0.360 / 0.271			0.317 / 0.273		

Supplementary Note 5: Effect of female size on reproductive output

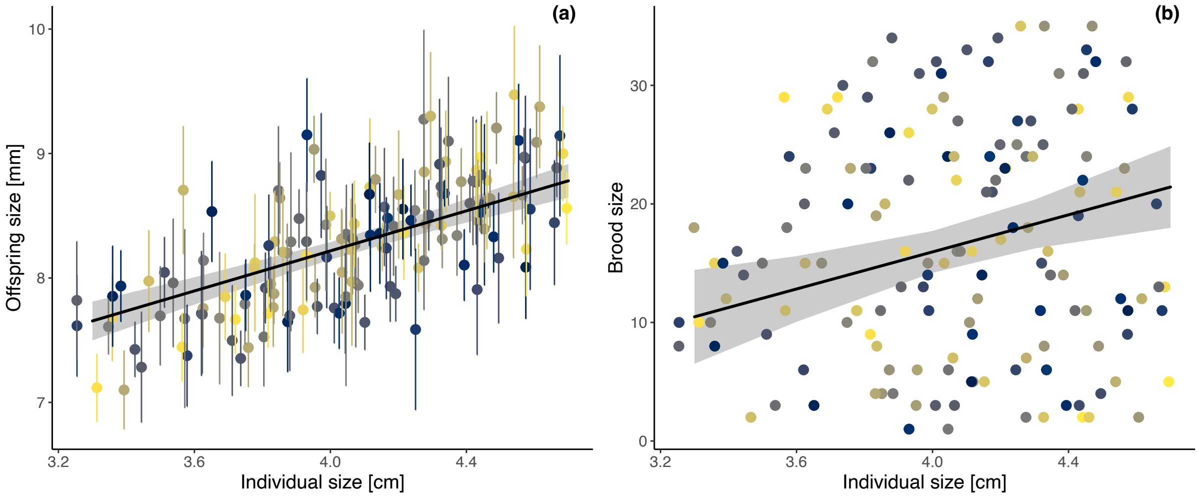

Supplementary Figure 2: Effects of female size at parturition on reproductive output. Female size at parturition (predicted from individual von Bertalanffy growth curves) is positively correlated with (a) offspring size (shown is mean offspring size (points) per brood ± SD (error bars), N = 2522 offspring from 144 broods) and (b) brood size (N = 152 broods). (a-b) Data points are colored by individuals, regression lines (black) with 95% confidence intervals (grey shadow) were estimated via linear mixed-effects models (**Supplementary Table 9**).

Supplementary Note 6: No effects of size at birth on behavior, reproduction, and growth

Supplementary Table 12 Full (left) and final (right) linear mixed-effect models testing if size on the first day of life predicts early-life behavior.

Response	*Predictor*	Full model			Final model		
		Estimate	*CI*	*P-value*	*Estimate*	*CI*	*P-value*
Activity [cm/sec]	Intercept	1.546	-1.112 – 4.204	-	0.996	0.577 – 1.416	-
	Size at birth [cm]	-0.656	-3.786 – 2.475	0.672	-	-	-
	Mother ID [m2]	1.210	0.301 – 2.118	0.025	1.188	0.298 – 2.077	0.025
	Mother ID [m3]	0.595	-0.079 – 1.269		0.524	-0.050 – 1.098	
	Observations	34			34		
	R^2 / R^2 adjusted	0.218 / 0.139			0.213 / 0.162		
Feeding [min]	(Intercept)	10.888	-38.316 – 60.093	-	48.058	42.847 – 53.268	-
	Size at birth [cm]	42.305	-15.642 – 100.252	0.146	-	-	-
	Mother ID [m2]	-9.241	-26.064 – 7.581	0.454	-	-	-
	Mother ID [m3]	1.021	-11.450 – 13.493		-	-	-
	Observations	34			34		
	R^2 / R^2 adjusted	0.151 / 0.066			0.000 / 0.000		

Supplementary Table 13 Full (left) and final (right) linear mixed-effect models testing if size on the first day of life predicts reproductive output.

Response	*Predictor*	Full model			Final model		
		Estimate	*CI*	*P-value*	*Estimate*	*CI*	*P-value*
Brood size	(Intercept)	-1.708	-18.716 – 15.300	-	7.187	1.217 – 13.157	-
	Size at birth [cm]	10.904	-8.548 – 30.356	0.270	-	-	-
	Age at parturition [weeks]	0.208	0.019 – 0.398	0.031	0.215	0.026 – 0.404	0.027
	Mother ID [m2]	4.675	-1.168 – 10.518	0.086	5.085	-0.828 – 10.999	0.011
	Mother ID [m3]	4.561	0.254 – 8.868		5.875	2.167 – 9.583	
	**Random effects**						
	σ^2	74.06			73.90		
	τ_{00}	8.19 Test fish ID			9.32 Test fish ID		
	ICC	0.10			0.11		
	N	34 Test fish ID			34 Test fish ID		
	Observations	152			152		
	Marginal R^2 / Conditional R^2	0.116 / 0.204			0.105 / 0.206		
Offspring size [mm]	Intercept	8.036	7.306 – 8.766	-	7.685	7.448 – 7.921	-
	Size at birth [cm]	-0.431	-1.276 – 0.414	0.315	-	-	-
	Age at parturition [weeks]	0.031	0.024 – 0.038	<0.001	0.031	0.024 – 0.038	<0.001
	Mother ID [m2]	-0.035	-0.288 – 0.218	<0.001	-0.053	-0.307 – 0.201	<0.001
	Mother ID [m3]	-0.462	-0.649 – -0.276		-0.514	-0.674 – -0.353	
	**Random effects**						
	σ^2	0.10			0.10		
	τ_{00}	0.02 Test fish ID			0.02 Test fish ID		
	ICC	0.18			0.19		
	N	34 Test fish ID			34 Test fish ID		
	Observations	144			144		
	Marginal R^2 / Conditional R^2	0.525 / 0.612			0.519 / 0.612		

Supplementary Table 14 Full (left) and final (right) linear mixed-effect models testing if size on the first day of life predicts the growth rate K and predicted final size L_{inf}.

Response	*Predictor*	Full model			Final model		
		Estimate	*CI*	*P-value*	*Estimate*	*CI*	*P-value*
L_{inf} [cm]	Intercept	4.171	3.473 – 4.870	-	4.716	4.602 – 4.831	-
	Size at birth [cm]	0.650	-0.172 – 1.473	0.117	-	-	-
	Mother ID [m2]	0.019	-0.220 – 0.258	<0.001	0.041	-0.202 – 0.284	<0.001
	Mother ID [m3]	-0.407	-0.584 – -0.230		-0.337	-0.493 – -0.180	
	Observations	34			34		
	R^2 / R^2 adjusted	0.472 / 0.419			0.426 / 0.389		
K	Intercept	0.309	0.268 – 0.350	-	0.305	0.263 – 0.346	-
	L_{inf} [cm]	-0.045	-0.054 – -0.036	<0.001	-0.047	-0.056 – -0.039	<0.001
	Size at birth [cm]	-0.017	-0.037 – 0.004	0.107	-	-	-
	Mother ID [m2]	0.006	-0.000 – 0.011	<0.001	0.005	-0.001 – 0.011	<0.001
	Mother ID [m3]	-0.016	-0.021 – -0.010		-0.018	-0.023 – -0.014	
	Observations	34			34		
	R^2 / R^2 adjusted	0.824 / 0.799			0.807 / 0.788		

Supplementary Note 7: Protocol for the 9-day behavioral assay phase

Immediately following the 28-day behavioral assay phase followed a period of nine days (starting on day 29) were individuals were assayed for three behavioral metrics: activity in a new tank, sociability, and response to a novel object. These data were collected for an auxiliary study that did not bear on the questions addressed in our article, following 3R guidelines to maximize data collected from individual animals. For the sake of experimental reproducibility and full transparency, we here report the full description and methodological details of these assays.

During this 9-day behavioral assay phase, individuals were assayed once a day, with a single behavioral assay conducted on each day (activity in a new tank, sociability, and response to a novel object), using the same automated, high-resolution recording system as during the preceding 28-day behavioral assay phase. The assays were conducted in the following order and then repeated two more times in the same order (i.e., any particular test was conducted a total of three times). On day 1, individuals were assayed for their activity in a new tank. On day 2, individuals experienced either a sociability test or a novel object test in randomized order. On day 3, individuals experienced whichever of the sociability or novel object tests that was not experienced on day 2.

To initiate the activity in a new tank assay, all tanks were cleaned and individuals were swapped among observation tanks. Activity (average distance moved per second, in cm) was tracked over a period of 60 minutes, starting one minute after transfer. For the following sociability and novel object tests, individuals remained in the same tank used for the activity in a new tank assay. Care was taken to ensure that individuals experienced each of a total of four tank systems (three systems in addition to the tank system in which they were kept for the first 28 days) as well as all possible tank positions (centre vs. periphery).

Sociability tests were conducted by introducing a round, clear acrylic container (10 cm diameter, 10 cm tall) containing two conspecifics into the tank. Conspecifics were matched to the size of stimulus fish as closely as possible (mean ± SD total length = 1.8 ± 0.3 cm; N = 80 randomly chosen from N = 176 stimulus fish used) but were otherwise randomly chosen from a stock tank. Each conspecific stimulus fish was used only once during the course of the experiment. Additionally, in order to control for a potential effect of the container itself, an empty container was also introduced to the tank, with the position of the empty container vs. the container containing conspecifics being randomized between two possible positions. The focal individual and the stimulus fish were given a 10-minute acclimation period, during which containers were surrounded with opaque white flower pots, followed by a 60-minute trial period (starting one minute after the flower pots were gently removed).

Novel object tests were conducted by introducing one of the following three novel objects to the tanks (such that every fish experienced each of the three objects once over the course of three novel object tests in a randomized order): a clear bounce ball filled with star-

shaped colorful glitter specks (red, green, yellow, blue; ball diameter = 4.3 cm); a ceramic, light green flower pot (diameter = 8 cm, height = 7 cm), and a plastic pink anemone (diameter = 10 cm).
