# Supplementary material for: Reproductive individuality of clonal fish raised in near-identical environments and its link to early-life behavioral individuality
Source: Nat Commun. 2023 Nov 24;14:7652. doi: 10.1038/s41467-023-43069-6 (PMC10673926; doi:10.1038/s41467-023-43069-6)
Supplement: Supplementary file 2 — Reporting Summary [file 41467_2023_43069_MOESM2_ESM.pdf]

nature portfolio

Corresponding author(s): Ulrike Scherer

Last updated by author(s): Oct 10, 2023

# Reporting Summary

Nature Portfolio wishes to improve the reproducibility of the work that we publish. This form provides structure for consistency and transparency in reporting. For further information on Nature Portfolio policies, see our Editorial Policies and the Editorial Policy Checklist.

## Statistics

For all statistical analyses, confirm that the following items are present in the figure legend, table legend, main text, or Methods section.

| n/a | Confirmed | |
|---|---|---|
| ☐ | ☒ | The exact sample size (*n*) for each experimental group/condition, given as a discrete number and unit of measurement |
| ☐ | ☒ | A statement on whether measurements were taken from distinct samples or whether the same sample was measured repeatedly |
| ☐ | ☒ | The statistical test(s) used AND whether they are one- or two-sided<br>*Only common tests should be described solely by name; describe more complex techniques in the Methods section.* |
| ☐ | ☒ | A description of all covariates tested |
| ☐ | ☒ | A description of any assumptions or corrections, such as tests of normality and adjustment for multiple comparisons |
| ☐ | ☒ | A full description of the statistical parameters including central tendency (e.g. means) or other basic estimates (e.g. regression coefficient) AND variation (e.g. standard deviation) or associated estimates of uncertainty (e.g. confidence intervals) |
| ☐ | ☒ | For null hypothesis testing, the test statistic (e.g. *F*, *t*, *r*) with confidence intervals, effect sizes, degrees of freedom and *P* value noted<br>*Give P values as exact values whenever suitable.* |
| ☒ | ☐ | For Bayesian analysis, information on the choice of priors and Markov chain Monte Carlo settings |
| ☐ | ☒ | For hierarchical and complex designs, identification of the appropriate level for tests and full reporting of outcomes |
| ☒ | ☐ | Estimates of effect sizes (e.g. Cohen's *d*, Pearson's *r*), indicating how they were calculated |

*Our web collection on statistics for biologists contains articles on many of the points above.*

## Software and code

Policy information about availability of computer code

| | |
|---|---|
| Data collection | All recordings of individual behavior were tracked using the software Biotracker Version 3.2.1 (link to the github repository https://github.com/BioroboticsLab/biotracker_core/wiki). We developed a custom-repository (publicly available on github: https://github.com/lukastaerk/Fish-Tracking-Visualization) to calculate daily activity and time spent feeding from Biotracker csv-files. |
| Data analysis | All data were analyzed and visualized using the software R (version 4.2.1). |

For manuscripts utilizing custom algorithms or software that are central to the research but not yet described in published literature, software must be made available to editors and reviewers. We strongly encourage code deposition in a community repository (e.g. GitHub). See the Nature Portfolio guidelines for submitting code & software for further information.

## Data

Policy information about availability of data

All manuscripts must include a data availability statement. This statement should provide the following information, where applicable:

- Accession codes, unique identifiers, or web links for publicly available datasets
- A description of any restrictions on data availability
- For clinical datasets or third party data, please ensure that the statement adheres to our policy

The data generated in this study have been deposited in Figshare [https://doi.org/10.6084/m9.figshare.23971599.v161. Source data are provided with this paper.

## Research involving human participants, their data, or biological material

Policy information about studies with human participants or human data. See also policy information about sex, gender (identity/presentation), and sexual orientation and race, ethnicity and racism.

| | |
|---|---|
| Reporting on sex and gender | N/A |
| Reporting on race, ethnicity, or other socially relevant groupings | N/A |
| Population characteristics | N/A |
| Recruitment | N/A |
| Ethics oversight | N/A |

Note that full information on the approval of the study protocol must also be provided in the manuscript.

# Field-specific reporting

Please select the one below that is the best fit for your research. If you are not sure, read the appropriate sections before making your selection.

[x] Life sciences [ ] Behavioural & social sciences [ ] Ecological, evolutionary & environmental sciences

For a reference copy of the document with all sections, see nature.com/documents/nr-reporting-summary-flat.pdf

# Life sciences study design

All studies must disclose on these points even when the disclosure is negative.

| | |
|---|---|
| Sample size | Sample size was determined based on previous experience gained in comparable studies (i.e., studies on the same species where we estimated the same parameter, namely repeatability) and based on recommendations for the field (Dingemanse & Dochtermann 2013, Bierbach et al. 2017, Laskowski et al. 2022).<br><br>Dingemanse NJ, Dochtermann NA (2013) Quantifying individual variation in behaviour: mixed-effect modelling approaches. Journal of Animal Ecology 82(1):39-54.<br>Bierbach D, Laskowski KL, Wolf M (2017) Behavioural individuality in clonal fish arises despite near-identical rearing conditions. Nature Communications 8:15361.<br>Laskowski KL, Bierbach D, Jolles J W, Doran C, Wolf M (2022) The emergence and development of behavioral individuality in clonal fish. Nature Communications 13(1):6419. |
| Data exclusions | For all test individuals, we collected behavioral and reproductive data. Analyses were performed on individuals for which we were able to collect complete behavioral and reproductive data for the observation period of 280 days. We excluded 7 individuals from our analyses for which we have no reproductive data; and another 4 individuals for which we have partial reproductive data only. These females were removed either because of technical issues, sickness, or death. |
| Replication | Our study does not include replication. |
| Randomization | Our study does not include any treatments or experimental groups. |
| Blinding | All test individuals were treated identically, i.e., we do not have treatments or experimental groups. Behavioral types and reproductive success of individuals were estimated after the practical procedures were completed avoiding any observation biases. |

# Reporting for specific materials, systems and methods

We require information from authors about some types of materials, experimental systems and methods used in many studies. Here, indicate whether each material, system or method listed is relevant to your study. If you are not sure if a list item applies to your research, read the appropriate section before selecting a response.

## Materials & experimental systems

| n/a | Involved in the study |
|---|---|
| ☒ | ☐ Antibodies |
| ☒ | ☐ Eukaryotic cell lines |
| ☒ | ☐ Palaeontology and archaeology |
| ☐ | ☒ Animals and other organisms |
| ☒ | ☐ Clinical data |
| ☒ | ☐ Dual use research of concern |
| ☒ | ☐ Plants |

## Methods

| n/a | Involved in the study |
|---|---|
| ☒ | ☐ ChIP-seq |
| ☒ | ☐ Flow cytometry |
| ☒ | ☐ MRI-based neuroimaging |

# Animals and other research organisms

Policy information about studies involving animals; ARRIVE guidelines recommended for reporting animal research, and Sex and Gender in Research

| | |
|---|---|
| Laboratory animals | We used newborn Amazon mollies, Poecilia formosa, originating from a laboratory breeding stock at the Humboldt University. |
| Wild animals | We did not use wild animals. |
| Reporting on sex | Our study species, the Amazon molly, is a naturally clonal all-female species. Therefore, all test individuals in this study were females. |
| Field-collected samples | Our study does not include samples collected from the field. |
| Ethics oversight | All animal care and experimental protocols complied with local and federal laws and guidelines and were approved by the appropriate governing body in Berlin, Germany, the Landesamt fur Gesundheit und Soziales (LaGeSo G-0224/20). |

Note that full information on the approval of the study protocol must also be provided in the manuscript.
